# The role of DHCR24 in the pathogenesis of AD: re-cognition of the relationship between cholesterol and AD pathogenesis

**DOI:** 10.1186/s40478-022-01338-3

**Published:** 2022-03-16

**Authors:** Xiaojing Bai, Meiting Mai, Kai Yao, Mengqi Zhang, Yue Huang, Wenbin Zhang, Xiaorou Guo, Yixuan Xu, Ying Zhang, Atikam Qurban, Lijie Duan, Jimei Bu, Jianfeng Zhang, Junfeng Wu, Yongfei Zhao, Xiangshan Yuan, Hengbing Zu

**Affiliations:** 1 Department of Neurology, Jinshan Hospital Affiliated to Fudan University, No. 1508 Long-hang Road, Jinshan District, Shanghai, 201508 China; 2 State Key Laboratory of Medical Neurobiology and Ministry of Education Frontiers Center for Brain Science, Institutes of Brain Science, Fudan University, Shanghai, 200032 China

**Keywords:** DHCR24, Cholesterol, Cholesterol deficiency, Pathogenesis, Hypothesis, Alzheimer’s disease

## Abstract

Previous studies show that 3β-hydroxysterol-Δ24 reductase (DHCR24) has a remarked decline in the brain of AD patients. In brain cholesterol synthetic metabolism, DHCR24 is known as the heavily key synthetase in cholesterol synthesis. Moreover, mutations of DHCR24 gene result in inhibition of the enzymatic activity of DHCR24, causing brain cholesterol deficiency and desmosterol accumulation. Furthermore, in vitro studies also demonstrated that DHCR24 knockdown lead to the inhibition of cholesterol synthesis, and the decrease of plasma membrane cholesterol and intracellular cholesterol level. Obviously, DHCR24 could play a crucial role in maintaining cholesterol homeostasis via the control of cholesterol synthesis. Over the past two decades, accumulating data suggests that DHCR24 activity is downregulated by major risk factors for AD, suggesting a potential link between DHCR24 downregulation and AD pathogenesis. Thus, the brain cholesterol loss seems to be induced by the major risk factors for AD, suggesting a possible causative link between brain cholesterol loss and AD. According to previous data and our study, we further found that the reduced cholesterol level in plasma membrane and intracellular compartments by the deficiency of DHCR24 activity obviously was involved in β-amyloid generation, tau hyperphosphorylation, apoptosis. Importantly, increasing evidences reveal that the brain cholesterol loss and lipid raft disorganization are obviously linked to *neuropathological* impairments which are associated with AD pathogenesis. Therefore, based on previous data and research on DHCR24, we suppose that the brain cholesterol deficiency/loss might be involved in the pathogenesis of AD.

## Introduction

**Fig. 1:**
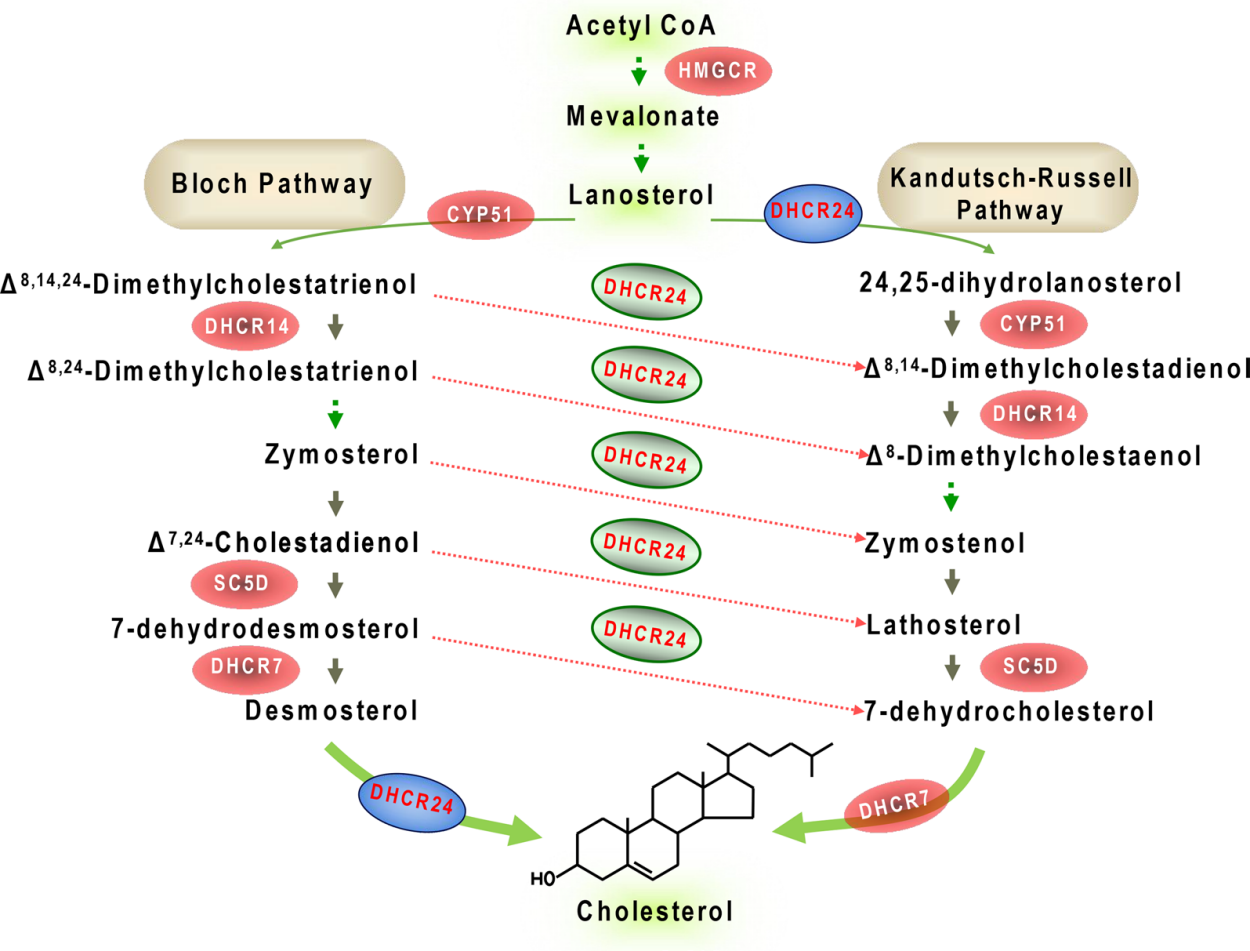
The critical role of DHCR24 in cholesterol synthesis and homeostasis. In the post-lanosterol pathway of cholesterol synthesis, the final step in the Bloch pathway or the first step in the Kandutsch–Russell pathway is catalyzed by the enzyme DHCR24. Besides, as a link bridge between two pathways, DHCR24 can theoretically act on any intermediate from lanosterol through to desmosterol to transfer intermediates from the Bloch to the K–R pathway. Thus, DHCR24 play the critical role in maintaining cholesterol homeostasis via the control of cholesterol synthesis

In 2000, Greeve et al. first found that there is a significant reduction in the expression of new gene in vulnerable brain regions in Alzheimer’s disease (AD) patients, which was named selective Alzheimer’s disease indicator 1 (Seladin-1), namely 24-dehydrocholesterol reductase (DHCR24) [45, 57]. In the post-lanosterol pathway of cholesterol synthesis, the final step in the Bloch pathway or the first step in the Kandutsch–Russell (K–R) pathway is catalyzed by the enzyme DHCR24 [132, 133]. Besides, as a link bridge between two pathways, DHCR24 can theoretically act on any intermediate from lanosterol through to desmosterol to transfer intermediates from the Bloch to the K–R pathway [30, 132]. Importantly, DHCR24 can also synergistically control the activity of 7-dehydrocholesterol reductase (DHCR7), a final key enzyme in the K–R pathway, which would ensure concerted control of cholesterol synthesis [85, 132]. From a physiological role, DHCR24 is universally regulated by sterols, dexamethasone, sex steroids, adrenocorticotropic hormone, thyroid hormone, Neurotrophins, and xenobiotics [22, 87, 112]. Furthermore, DHCR24 activity is also regulated by ubiquitination, phosphorylation, and epigenetic factors such as methylation and acetylation [64, 87]. Collectively, accumulating evidences suggest that the modulation of DHCR24 activity could be a key node in the control of cholesterol synthesis. To sum up, above findings reveal that DHCR24 could play a crucial role in maintaining the cholesterol homeostasis via the control of cholesterol synthesis (Fig. 1).

**Fig. 2:**
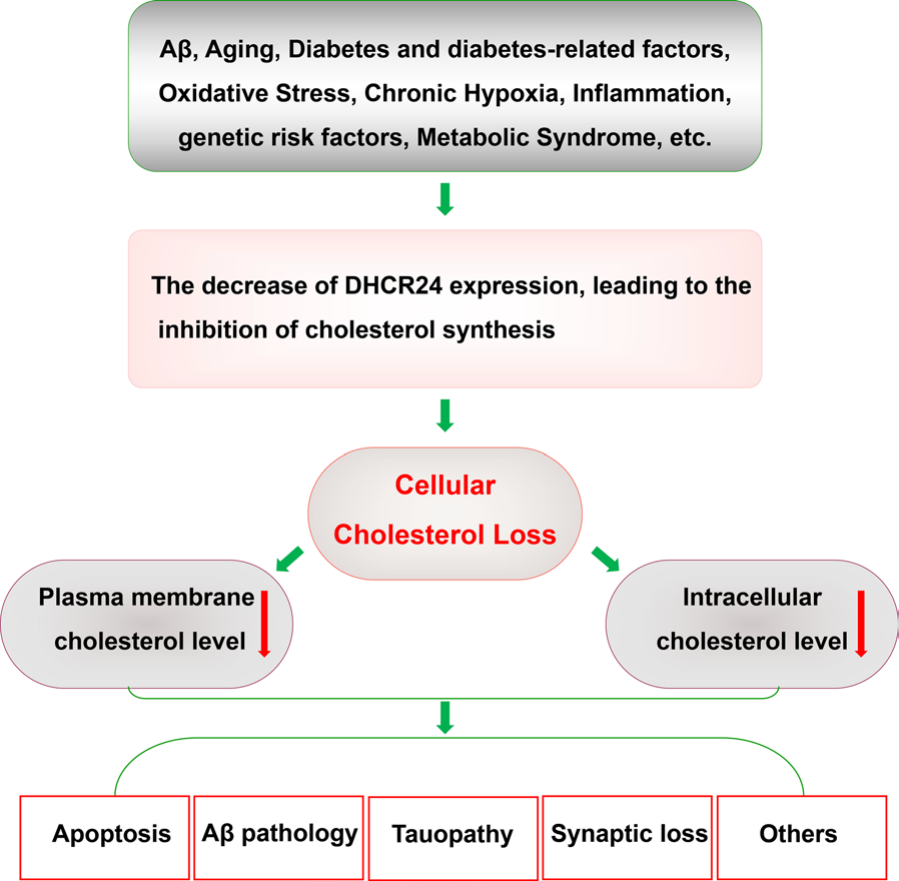
The contribution of DHCR24 to Alzheimer’s disease. The downregulation of DHCR24 could be induced by risk factors from FAD and SAD, including Aβ, aging, diabetes-related risk factors, chronic hypoxia, oxidative stress, chronic inflammation, insufficiency of brain neurotrophic substances, and metabolic syndrome, suggesting a causative link between DHCR24 downregulation and major risk factors from AD. Furthermore, DHCR24 downregulation lead to the inhibition of cholesterol synthesis and decrease of cholesterol level in the plasma membrane and intracellular organelles, resulting in cholesterol deficiency-induced pathological impairments, such as Aβ overproduction, tau hyperphosphorylation, apoptosis, synaptic impairment, and other pathological impairments, which are associated with neurodegenerative diseases such as AD. Thus, the downregulation of DHCR24 could contribute to Alzheimer’s disease and other neurodegenerative diseases

Nevertheless, DHCR24 activity is also obviously downregulated by major risk factors from AD, such as aging, diabetes-related factors, amyloid-β (Aβ), oxidative stress, chronic inflammation, and genetic factors [11, 20, 42, 55, 61, 63, 70, 107, 125, 135]. Thus, the above data suggest that downregulation of DHCR24 is obviously linked to the major risk factors from AD, suggesting a potential causative link between DHCR24 downregulation and major risk factors from AD. Moreover, a growing body of research has shown that deficiency of DHCR24 activity could induce lowering cholesterol level of neuronal cells and disruption of membrane lipid-raft structure and function, leading to the disregulation of cellular cholesterol homeostasis, and abnormality of cell signaling [3, 9, 24, 71, 122]. In addition, increasing evidences support that downregulation of DHCR24 could lead to Aβ production, apoptosis of neuronal or glial cells, hyperphosphorylation of microtubule-associated protein tau (tau), inhibition of autopagy, and inflammation, which are tightly associated with AD and other degenerative diseases [9, 24, 45, 71, 83, 95, 127, 155]. Therefore, previous studies strongly support that that the reduced cholesterol level in plasma membrane and/or intracellular compartments by the deficiency of DHCR24 obviously contributes to neurodegeneration such as AD (Fig. 2).

**Fig. 3:**
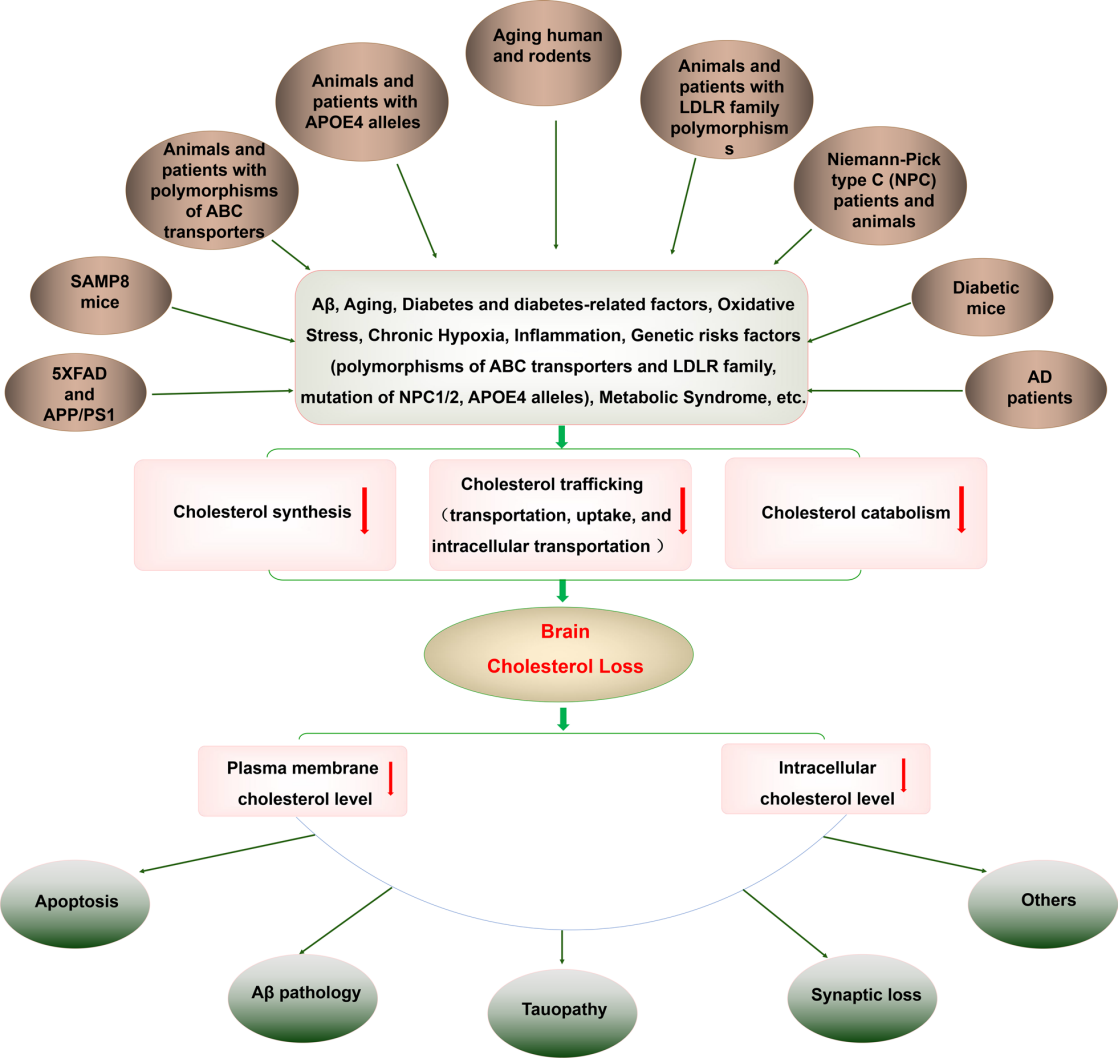
A Revised Cholesterol Hypothesis of AD. There are abnormal alterations in brain cholesterol metabolism, including the decrease of de novo cholesterol synthesis, and/or cholesterol trafficking (transportation, uptake, and intracellular transportation), and/or cholesterol catabolism in aging humans and animals, SAMP8 mice, diabetic mice, FAD (5xFAD and APP/PS-1) animals, AD patients, genetic forms of AD animals and patients (ApoE4 allele, mutation of NPC1 or NPC2, polymorphism of ABC and LDL receptor family), suggesting the brain cholesterol insufficiency/loss. To some extent, we found that these different kinds of AD models and patients include major risk factors for AD, such as Aβ, genetic factors, aging, diabetes-related risk factors, chronic hypoxia, oxidative stress, chronic inflammation, and metabolic syndrome, etc. Thus, the brain cholesterol loss seems to be induced by the major risk factors for AD in these different kinds of AD models and patients, suggesting a possible causative link between brain cholesterol loss and AD. Importantly, the brain cholesterol loss might lead to the membrane lipid raft disorganization and decrease of intracellular compartments, resulting in the pathological impairments which are associated with AD pathogenesis. Therefore, based on previous data and research on DHCR24, we suppose that the brain cholesterol deficiency/loss might be involved in the onset and progression of AD

In addition, a growing body of evidence reveals that there are abnormal alterations in brain cholesterol metabolism, including the decrease of de novo cholesterol synthesis, and/or cholesterol trafficking (transportation, uptake, and intracellular transportation), and/or cholesterol catabolism, in aging human and mice, senescent-accelerated mice strain 8 (SAMP8) mice, diabetic mice, familial Alzheimer's disease (FAD) mice, genetic forms of AD animals and patients, and AD patients, suggesting the brain cholesterol loss [15, 18, 54, 66, 72, 89, 92, 107, 110, 111, 123, 129, 142, 146, 152]. The brain cholesterol loss appears to be a pervasive, prominent and common feature in these different kinds of AD models and patients. To some extent, we found that these different kinds of AD models and patients include major risk factors for AD, such as Aβ, genetic factors, aging, diabetes-related factors, chronic hypoxia, oxidative stress, chronic inflammation, and metabolic syndrome, etc. The brain cholesterol loss seems to be tightly associated with major risk factors from AD. Thus, we suppose that the brain cholesterol loss is likely to be induced by the major risk factors for AD, suggesting a possible causative link between brain cholesterol loss and AD (Fig. 3). Surprisingly, accumulating data also reveal that the brain cholesterol loss is very likely to occur in the initiation stage of AD pathology, suggesting a key role of brain cholesterol loss in initial changes of AD pathogenesis [15, 18, 28, 34, 35, 80, 92, 110, 111, 124, 141, 150]. Furthermore, the brain cholesterol deficiency seems to be an early and common driving factor in the onset and development of AD. And the brain cholesterol deficiency could be intimately linked with the generation of β-amyloid, tauopathy, synaptic loss, neuronal apoptosis and death, which are associated with the pathogenesis of AD [5, 9, 21, 24, 45, 73–75, 93, 95, 115]. Based on previous data and research on DHCR24, we suppose that the brain cholesterol deficiency/loss could trigger the onset and progression of AD.

## The critical role of DHCR24 in cholesterol synthesis and homeostasis

The cholesterol synthesis pathway encompasses more than 20 enzymes and can be divided into the early sterol synthesis pathway and the post-lanosterol pathway [132, 133]. In the post-lanosterol pathway, the pathway can take one of two intertwined routes, the Bloch and K–R pathway [132, 133], creating a long and complex road to cholesterol through various branch points. Theoretically speaking, DHCR24 might be a key synthetase heavily involved in cholesterol synthesis (Fig. 1).

Firstly, in the post-lanosterol pathway, lanosterol can be acted upon by Lanosterol 14-a-demethylase (LDM or CYP51A1) to enter the Bloch pathway [132, 133]. In the Bloch pathway, by reducing the double bond at carbon 24 of the last cholesterol precursor, desmosterol, the final step in the Bloch pathway is catalyzed by the enzyme DHCR24 [30, 132, 157]. And lanosterol can be also acted upon by DHCR24 to enter the K–R pathway, so DHCR24 can also control the gate of entry in the K–R pathway [30, 132, 157]. Besides, DHCR24 can theoretically act on any intermediate from lanosterol through to desmosterol to transfer intermediates from the Bloch to the K–R pathway, so it is also a link bridge between two pathways [30, 132]. Furthermore, in post-squalene pathways, DHCR7 is another important cholesterol synthetase, which controls the final step of the K–R pathway [30, 85, 132]. A previous study reveals that when the DHCR24 gene is knocked down, DHCR7 activity is also ablated. Conversely, overexpression of DHCR24 enhances DHCR7 activity [85]. So, DHCR7 activity obviously is controlled by DHCR24, which would ensure concerted control of cholesterol synthesis [85, 132]. Thus, DHCR24 obviously control the cholesterol synthesis in the post-lanosterol pathway.

In addition, mutations in DHCR24 enzyme, which converts desmosterol into cholesterol, leads to desmosterolosis, an autosomal recessive developmental disorder [3, 122, 128]. Defect in the enzyme DHCR24 causes significant elevation of the cholesterol precursor desmosterol and cholesterol deficiency [3, 6, 122]. Moreover, in DHCR24 knockout (KO) mice the brain cholesterol lack with age, and brain cholesterol deficiency in 3-week-old was associated with altered membrane composition including disrupted detergent-resistant membrane domain (DRM) structure [3, 71]. Similarly, in silencing DHCR24 cell model, it was found that cell desmosterol and 7-dehydrocholesterol (7-DHC) are significantly elevated, and cell cholesterol is greatly decreased [9, 24, 127]. So, this genetic defect manifests that the defect of DHCR24 enzyme activity leads to cholesterol deficiency, suggesting the critical role of DHCR24 in maintaining cholesterol synthesis and homeostasis.

Furthermore, the posttranslational phosphorylation modification of DHCR24 has been identified by cell kinase signals, which is a major mode of regulating cholesterol homeostasis [86, 87]. Moreover, data have identified particular putative phosphorylated sites on DHCR24, such as T110, Y299, Y321, and Y507 [87]. In addition, protein kinase C (PKC) ablated DHCR24 activity by inhibiting a major serine/threonine kinase [87]. Thus, modulating DHCR24 activity by phosphorylation would allow for a rapid means of regulating cholesterol synthesis. In addition to phosphorylation modification, a lot of evidences supported that DHCR24 could be ubiquitinated, and 11 ubiquitination sites are identified, suggesting that DHCR24 activity may be regulated by ubiquitin-proteasomal degradation [64, 87]. To sum up, these studies indicate two important regulatory mechanisms for DHCR24 activity by cell kinase signals-mediated phosphorylation and by ubiquitin-proteasomal degradation.

As mentioned above, accumulating evidence indicates that DHCR24 play the critical role in maintaining cholesterol homeostasis via the control of cholesterol synthesis (Fig. 1). According to the above data, we conclude that the modulation of DHCR24 activity could be a key node in the control of cholesterol synthesis and homeostasis.

## A causative link between DHCR24 downregulation and risk factors from AD

### Amyloid-β proteins

In the amyloid cascade theory, amyloid-β protein (Aβ) is regarded as a key risk substance, which is tightly related to FAD and partly to sporadic AD (SAD) [8, 29]. In Neuro-2A cells, to be combined exposure of amyloid-β peptide 1–40 (Aβ40) or amyloid-β fragment 25–35 (Aβ25–35), the expressions of seladin-1 genes were significantly down-regulated [135]. Moreover, in vitro C6 astrocytic cell lines, we also confirmed that Aβ40 or Aβ25–35 could markedly induce the downregulation expressions of seladin-1 [14]. Additionally, Najem et al. also found amyloid-β peptide 1–42 (Aβ42) could induce the downregulation of DHCR24 and inhibited cholesterol synthesis pathway in SH-SY5Y cells [102]. Thus, above findings suggest that the downregulation of DHCR24 expression could be induced by β-amyloid proteins.

In AD patients, it has been reported that DHCR24 transcription and protein expression were selectively down-regulated in the brain areas affected in Alzheimer's disease, but the reasons for this decrease are not known [45, 57]. In APPswe/PS1deltaE9 (APP/PS1) AD mice, Vanmierlo et al. found that reduced expression of DHCR24 gene in both cortex and cerebellum as aging [57, 151].

Besides, in APP/PS1 mice, the decreased cholesterol level and increased phospholipids/cholesterol ratio might lead to the disruption of lipid raft homeostasis, which has been considered to contribute to cellular deregulation, resulting in neuronal loss in AD [35]. Further analysis found that the change of lipid raft alteration occurred in the early stage of AD pathology in APP/PS1 mice [35]. Noticeably, in 5xFAD and APP/PS1 mice brain, why is there the inhibition of cholesterol biosynthesis at the very early stage of AD? Park et al. found that Aβ production might directly correlate with cholesterol biosynthesis inhibition [110]. Furthermore, some early studies show that Aβ40/42 inhibits cholesterol synthesis and reduced cellular cholesterol levels in neuronal or glial cells by inhibiting the main cholesterol biosynthesis enzymes [14, 43, 46, 102, 135]. Intriguingly, in FAD mice, the initial increase in the production of Aβ is mutations-based and occurs relatively early, Aβ overload might induce the downregulation of cholesterol synthetic genes, resulting in the brain cholesterol loss, which could also occur in the initial stage of FAD. Does overproduction of beta-amyloid also trigger a cholesterol loss cascade leading to neurodegeneration in the early stage of FAD? In fact, APP transgenic mice exhibited lower levels of cellular cholesterol in their brains, and conversely, APP knockout mice exhibited higher levels of cellular cholesterol in their brains, suggesting that Aβ mediated regulation of cellular cholesterol synthesis [148]. Collectively, above data suggest that Aβ overproduction is likely to be a risk factor for cholesterol biosynthesis. Taken together, accumulating data support that β-amyloid proteins could lead to the downregulation of cholesterol synthetic genes, including DHCR24.

### Diabetes and diabetes-related risk factors

Studies demonstrate that Diabetes mellitus (DM) enhances the risk for Alzheimer's disease [8, 13]. Moreover, diabetes-related risk factors, such as hyperglycemia, insulin insufficiency, and insulin resistance have been proposed to contribute to AD pathogenesis [13, 50, 145]. Kazkayasi et al. confirmed that constant lack of insulin for 5 days decreased DHCR24 levels in rat primary cultured neurons [61]. In addition, the intermittent high glucose concentrations also reduced the expression of DHCR24 in the human fetal neuroepithelial cells [42]. Besides, a decrease in DHCR24 was also found in the brains of rodents with streptozotocine (STZ)-induced diabetes [55, 61, 142]. Furthermore, in diabetic mice model, insulin as a regulator of DHCR24, the lack of insulin can downregulate the expression of all cholesterol synthetase, including sterol regulatory element-binding protein 2 (SREBP2), DHCR24 [61, 102, 142]. To sum up, all the above evidence indicates that the DM-related risk factors can induce the downregulation of DHCR24.

### Insufficiency of neurosteroid and other neurotrophic factors

Basic and clinical evidence suggests that neurosteroid such as estrogens and androgen, and neurotrophic factors such as insulin-like growth factors (IGFs) and neurotrophins, have protective effects in the brain [17, 112]. Nevertheless, their potential role against neurodegenerative diseases, in particular Alzheimer's disease, is still a matter of debate. Accumulating data demonstrated that neurosteroid could induce the expression of DHCR24, as well as the synthesis of cell cholesterol, in neurons and astrocytes [14, 17, 112, 160]. Similarity, thyroid hormones (TH) play an important role in the development of human brain, by upregulating the expression of specific DHCR24 genes in neuronal precursors [10]. Moreover, it has been found that IGF1 and nerve growth factor (NGF) induced upregulation of DHCR24 expression, and conversely, the inhibition of IGF signaling downregulated the expression of DHCR24 [22, 42]. On the contrary, dexamethasone could obviously decrease the expression of genes involved in cholesterol synthesis genes, such as squalene epoxidase (SQLE) and DHCR24 [58]. Besides, with aging, there is a progressive, age-dependent decline in the level of many important neurotrophic factors, such as estrogen, androgen, insulin, NGF and IGFs, in the brain of aged rodents and AD patients [19, 22, 112]. Thus, evidences suggest that the downregulation of DHCR24 induced by the depletion of neurosteroids and neurotrophic factors in the brain might play a pivotal pathological role in neurodegenerative diseases. In brief, these studies suggest that insufficiency of brain neurotrophic substances might lead to the decrease of DHCR24 expression in the brain, which is involved in maintaining cholesterol synthesis and homeostasis.

### Chronic hypoxia, oxidative stress and inflammation

Increasing data underscore the importance of chronic oxidative stress and inflammation in the pathogenesis of neurodegenerative diseases, including AD [23, 97, 136]. Kuehnle et al. show that DHCR24 expression is up-regulated in an acute response; conversely, upon chronic exposure to oxidative stress, the level of DHCR24 expression was lowered in SH-SY5Y cells [70, 125]. Moreover, in the hypoxia rat model, chronic hypoxia significantly induced the decrease of DHCR24 expression in the hippocampus [88]. In addition, Khuda et al. found that LPS-Induced inflammation obviously reduced the DHCR24 expression [63]. Collectively, under the pathological condition, chronic hypoxia, oxidative stress and inflammatory response could negatively modulate DHCR24 expression.

### Aging and metabolic syndrome

Aging, obesity and metabolic syndrome is a cluster of risk factors that participate in the development of neurodegenerative diseases such as AD [12, 76, 139]. Interestingly, a study on bariatric surgery was performed in order to investigate whole blood gene expression profiles in obese subjects that have obvious overweight, BMI abnormality, and insulin resistance problems, including some metabolic syndrome risk factors, the study showed that expression of DHCR24 was significantly decreased [11]. Additionally, all enzyme genes in the cholesterol synthesis pathway are significantly downregulated in the aging mice brain, such as hydroxy-3-methylglutaryl-CoA reductase (HMGCR), SQLE, 7-dehydrocholesterol reductase (DHCR7), and DHCR24, compared to the adult mice [11, 106, 107]. Thus, the above dada support that aging and metabolic syndrome-related risk factors could obviously downregulate the expression of DHCR24.

### Epigenetic factors

Cumulating Evidences suggest that epigenetic factors that are involved in Late-Onset Alzheimer's Disease (LOAD), such as methylation and acetylation, also obviously regulate DHCR24 expression and activity [26, 88]. Regarding epigenetic modifications, the up-to-date epigenomic findings include reported modifications in the LOAD core pathology loci DHCR24 [88]. Another epigenome-wide association study on obesity-related traits found that the novel DNA methylations were located on the DHCR24 [26, 31]. Thus, epigenetic modification could regulate DHCR24 gene expression which contributes to Late-Onset Alzheimer's Disease.

As stated above, increasing evidence reveals that the downregulation of DHCR24 could be induced by a lot of risk factors from AD, including Aβ, aging, diabetes-related factors, hypoxia, oxidative stress, chronic inflammation, insufficiency of brain neurotrophic substances, and metabolic syndrome, etc. Intriguingly, above data obviously suggests a causative link between DHCR24 downregulation and major risk factors from AD (Fig. 2). Therefore, we propose that the downregulation of DHCR24 might be an early and common regulatory pathway in the pathogenesis of FAD and SAD, which may be tightly associated with dysregulation of cholesterol homeostasis.

## DHCR24 downregulation and pathological impairments related to AD

### DHCR24 downregulation and Aβ metabolism

In recent years, in silencing DHCR24 SH-SY5Y cells, Sarajärvi et al. confirm that the reduced DHCR24 expression results in enhanced Golgi-localized gamma-ear-containing ARF binding protein 3 (GGA3) depletion, to further lead to augmented post-translational stabilization of beta-site amyloid precursor protein cleaving enzyme 1 (BACE1) and increased beta-amyloidogenic processing of amyloid precursor protein (APP) and Aβ production [127]. In addition, DHCR24-deficient mice brains had reduced levels of cholesterol and disorganized cholesterol-rich membrane lipid raft, leading to membrane-dependent plasmin inactivation and the displacement of β-secretase (BACE1) from membrane lipid-raft to APP-containing membrane fractions, to increased β-cleavage of APP and high levels of Aβ production [24, 71]. Thus, the above findings suggest that DHCR24 knockdown promotes the cleavage of APP and production of Aβ through the decrease of cholesterol levels and the reorganization of lipid raft. Altogether, these data suggest that the decrease of neuronal membrane cholesterol or intracellular cholesterol might contribute to excessive Aβ production.

### DHCR24 downregulation and tauopathy

Interestingly, in APP/PS1 transgenic animals, it is found that that the downregulation of seladin-1 expression in vulnerable AD brain areas is paralleled by an increase in the amount of hyperhosphorylated microtubule-associated protein tau (tau) [57]. Thus, Iivonen et al. suppose that the downregulation of DHCR24 expression might be associated with hyperphosphorylated tau in AD. Furthermore, in our study, after silencing DHCR24 by lentivirus-mediated DHCR24 short hairpin RNA (shRNA) in SH-SY5Y cells, we found silencing DHCR24 could markedly induce hyperphosphorylation of tau at some specific sites, including Thr181, Ser199, Thr231, Ser262, Ser396, and Ser422 [9, 119]. Besides, we further found that DHCR24 knockdown lead to the decrease of plasma membrane cholesterol and disruption of lipid raft/caveolae, resulting in inhibition of lipid raft-dependent phosphoinositide 3-kinase (PI3-K)/ protein kinase B (Akt) signaling, Protein phosphatase 2A (PP2A) signaling, as well as the overactivation of glycogen synthase kinases-3beta (GSK3β) and mammalian target of rapamycin (mTOR) signaling [9, 103, 119]. Similarly, defects in the cholesterol trafficking are associated with enhanced generation of hyperphosphorylated Tau and Amyloid-β protein [131]. Moreover, previous studies confirm that these sites are tightly correlated with a possible toxic effect of phosphorylated tau, which is involved in AD and other tauopathies [2, 4, 104]. Overall, these data suggest that cholesterol loss by DHCR24 knockdown could play a crucial role in tau hyperphosphorylation.

### DHCR24 downregulation and synaptopathy

Desmosterolosis is caused by mutations in DHCR24, lead to the elevated desmosterol levels and decreased level of cholesterol in the patient's brain, resulting in multiple congenital anomalies including white matter atrophy and synaptic abnormality [3, 71, 122, 159]. Furthermore, in a mouse model of desmosterolosis, DHCR24-KO mice brains showed complex changes in expression of lipid and sterol transcripts and synaptic plasticity transcripts, and the decrease of membrane cholesterol and disruption of membrane lipid raft and increased arborization synapse [3, 24, 71, 122]. On the contrary, the overexpression of DHCR24 significantly increased the total number of dendritic spines and the mushroom spines in mature mouse hippocampal neurons, facilitating synapse formation [95]. Very importantly, a body of evidences support that cholesterol reduction can trigger dysfunction of synaptic structure and function, and possible mechanisms by which cholesterol content in the plasma membrane influences synaptic processes [69, 78, 114]. Therefore, the cholesterol loss by DHCR24 downregulation may impair synapse formation, maturation, and function.

### DHCR24 downregulation and apoptosis

AD is characterized by severe neuronal and/or glial cells loss; however, the mechanisms by which neurons or glial cells die remain elusive [20, 105]. A line of studies has shown that over-expression of DHCR24 protected the cells from apoptotic cell death by amyloid-β-mediated toxicity or other stresses, and low-expression of DHCR24 obviously induced an apoptosis upon exposure to different stress conditions [45, 70, 82–84, 95, 127, 155]. Thus, these findings support that cholesterol deficiency by DHCR24 knockdown leads to a cell apoptosis under different pathological or stress conditions.

### DHCR24 downregulation and other pathological injuries

In our study, we found that DHCR24 knockdown could obviously induce the inhibition of autophagy [9]. In addition to Aβ pathology, tauopathy, synaptopathy, and apoptosis, thus, we suppose that cholesterol loss by DHCR24 knockdown also might be involved in other pathological injuries which are related to AD, such as autophagy, mitochondrial injuries, inflammation, neurosteroid synthesis, and other metabolic abnormalities. Certainly, further study is still to be performed in order to elucidate to complex relationship between DHCR24 and pathological impairments.

In conclusion, based on current knowledge about DHCR24, accumulating data support that there is an obvious link between DHCR24 downregulation and major risk factors from FAD and SAD (Fig. 1). Furthermore, compelling evidences support the deficiency of DHCR24 activity lead to the inhibition of cholesterol synthesis and decrease of cholesterol level in the plasma membrane and intracellular organelles, coupled with disruption of membrane lipid raft, resulting in cholesterol deficiency-induced pathological impairments [9, 45, 70, 82–84, 95, 112, 113, 127, 155]. Thus, accumulating evidences strongly reveal that cholesterol loss by DHCR24 downregulation could lead to Aβ overproduction, tau hyperphosphorylation, and other pathological impairments which are associated with neurodegenerative diseases such as AD (Fig. 2). Regretfully, because desmosterolosis by DHCR24 mutation is a lethal disorder, the mice model lacking one or both alleles of DHCR24 gene is still lack [3]. So, we still need to further investigate the role of DHCR24 in AD pathogenesis in in vitro or in vivo model systems.

## Alteration of cholesterol metabolism in different kinds of AD models and patients

### Alteration of cholesterol metabolism in aging humans and rodents, SAMP8 mice, and diabetic mice

Age-related brain aging is regarded as a major risk factor in the initiation and progression of Alzheimer's disease [98, 106, 130]. Compared to the adult mice, all enzyme genes in the cholesterol synthesis pathway are significantly downregulated in the aging brain, such as HMGCR, SQLE, DHCR7, and DHCR24, suggesting decreased de novo cholesterol biosynthesis in the aging brain [15, 106, 107]. On the contrary, genes involved in cholesterol-transporting proteins such as Apolipoprotein E (ApoE), are obviously upregulated, suggesting a compensatory response due to the decrease of cholesterol synthesis and decreased cholesterol level in the aging brain [15, 107]. Moreover, aging shows an age-dependent decrease of cholesterol level, and is accompanied by the decrease of synaptic cholesterol levels in the hippocampus [138]. This is consistent with the cholesterol loss observed in the cortex of aged rodents and humans [91, 92, 137, 138, 143]. However, others have found that although cholesterol synthesis is decreased in the hippocampus, the total brain cholesterol content remains stable [138, 146]. Thus, the reduction of cholesterol in the brain could present regional specificity during aging. Similarly, in SAMP8, it has been found that the hippocampus of SAMP8 mice presents reduced cholesterol levels at 6 months of age [111]. Further, although cholesterol levels did not differ in 2-month-old mice, a significant 35% decrease was observed in hippocampal extracts from 6-month-old SAMP8 mice [111]. The extent of the change was similar to that observed in the hippocampus of aged mice compared with young wild-type mice [91, 92, 138]. Pérez-Cañamás et al. confirm that cholesterol loss in the hippocampus of SAMP8 mice is an aging hallmark directly involved in cognitive decline. Overall, above data supports that these aging-related risk factors could induce the decrease of brain cholesterol synthesis and cholesterol level.

In recent years, it is confirmed that there is a significant reduction in expression of SREBP-2, and its downstream cholesterol synthesis genes in the diabetic mice brain, leading to a reduction in brain cholesterol synthesis in type 1 and type 2 diabetic mice [72, 91, 92, 124, 138]. Moreover, altered insulin signaling also modulates the expression of molecules involved in cholesterol biosynthesis, resulting in inhibition of brain cholesterol synthesis and decreased level of free cholesterol in diabetic mice brain [68, 72, 142]. And the lowering-expression of cholesterol synthesis genes is due, at least in part, to diabetes-related risk factors, such as insulin-deficiency, insulin resistance, lower or high glucose [72, 91, 92, 124, 138]. Thus, inhibition of brain cholesterol synthesis and cholesterol insufficiency could be induced by diabetes and diabetes-related risk factors.

### Alteration of cholesterol metabolism in 5xFAD and APP/PS-1 mice

In a recent study, Ye and colleagues found that in vitro cultured primary astrocytes stimulated with Aβ exhibited higher expression of ABC transporters that is involved in cholesterol efflux. Unsurprisingly, detection of this sterol revealed that the intracellular cholesterol level was significantly reduced in astrocytes [156]. The same expression pattern of ABC transporters was also found in 5xFAD mice, an AD mice model with early onset of Aβ pathology [156]. Park et al. confirmed that the majority of genes involved in cholesterol biosynthesis are obviously dysregulated in 5xFAD mice, suggesting the inhibition of the cholesterol biosynthesis genes and decrease of cholesterol biosynthesis in mice brain [110, 156]. Similarly, the main cholesterol synthetic genes were markedly downregulated in AD astrocytes of APP/PS1 [106, 107]. Conversely, further analysis found that main genes which mediate cholesterol transportation were significantly upregulated, such as apoE, ATP binding cassette A1 (ABCA1), low-density lipoprotein receptor (LDLR), and sterol O-acyltransferase 1 (SOAT1) in the APP/PS1 and 5xFAD mice brain, suggesting an increasing ability of cholesterol trafficking in order to compensate for cholesterol loss by decreasing cholesterol synthesis [107, 110, 156]. What's more, Park et al. also found that cholesterol synthetic genes were downregulated in FAD mice brain as a consequence of the chronically stimuli, such as Aβ, which is consistent with the previous studies [14, 43, 46, 102, 107, 110, 135, 148, 156]. Therefore, above data suggest that Aβ could lead to the inhibition of the cholesterol synthesis and the decrease of brain cholesterol in FAD mice brain.

### Alteration of cholesterol metabolism in AD patients

Intriguingly, many evidences suggest that de novo synthesis of cholesterol in the brain decline in AD patients [68, 117, 123, 129, 146]. In addition, compared with non-demented controls, the cerebrospinal fluid (CSF) levels of cholesterol and its precursors (lanosterol, lathosterol and desmosterol) are lower in the brain of AD patients, suggesting that cholesterol de novo synthesis within the brain of AD patients might be reduced [68]. And cholesterol synthesis is decreased in the hippocampus, while absolute cholesterol content remains at a stable level in the AD, suggesting a brain region-specific decrease of cholesterol synthesis [92, 123, 146]. Furthermore, in AD, the level of cholesterol is reduced in the hippocampus, lipid raft fraction in the whole brain, and white matter, coupled with membrane lipid structure perturbation; the brain cholesterol deficit/loss play a major role in the disruption of AD membrane lipid structure [1, 96, 101]. Consequently, above data suggest that the human AD brain could have decreased cholesterol levels, and a region-dependence of cholesterol synthesis is influenced [123, 129, 146]

Intriguingly, in a new study, Varma et al. found that the majority of genes (14/15) within the de novo cholesterol biosynthesis pathway, including 3 in pre-squalene and 12 in post-squalene, showed significantly lower gene expression in the entorhinal cortex and hippocampus of AD patients compared to the non AD control, and no alterations were detected in the visual cortex, suggesting a regional-specific reduction of cholesterol biosynthesis [152]. Very importantly, these alterations of differential and region-specific genes expression in the entorhinal cortex and hippocampus appears to provide insights into cholesterol homeostasis dysregulation in AD pathogenesis, which might be tightly related to the initiation and progression of AD [62, 152]. Notably, Varma et al. found that gene expression alterations identified in AD brain were not observed in PD brain, suggesting that these changes may be specific to AD. Thus, the author supposes that the decrease of brain cholesterol synthesis likely reflects fundamental features of AD pathogenesis [152].

In addition, it is striking that gene expression of cytochrome P450 46A1 (CYP46A1), is also significantly lowered in the entorhinal cortex and hippocampus in AD. Inactivation of CYP46A1 has been shown to lower cholesterol efflux from the brain, leading to a compensatory response due to the decrease in de novo cholesterol biosynthesis [152]. Furthermore, Varma et al. also show that the principal cholesterol precursor lanosterol and catabolic product 24S-hydroxycholesterol (24-OHC) is lower in AD, suggesting that both de novo cholesterol biosynthesis and catabolism are impaired by the disease [152]. Interestingly, a growing bulk of evidence reveals that CYP46A1 expression and its catabolic product 24-OHC content significantly decreased in late AD compared to control and early AD brains [41]. However, a few studies suggest that the increase of Cyp46A1 activity might be partly responsible for cholesterol loss in aged and AD brain [138]. Collectively, the decrease of CYP46A1 expression in AD brains is likely to be due to the compensatory response to brain cholesterol loss, or a selective loss of neurons expressing the enzyme CYP46A1 during AD development [41, 152]. Therefore, increasing evidence suggest that there is a decrease of the cholesterol biosynthesis and/or decrease of cholesterol catabolism, resulting in brain cholesterol loss in AD brain.

### Alteration of cholesterol trafficking in animals/patients with genetic forms of AD

The apoE4 allele is the dominant genetic risk factor for late-onset AD, and apoE4 has great influence in Aβ aggregation and clearance, tau pathogenesis, neuroinflammation, synaptic dysfunction, and neuronal loss [56, 89, 147]. However, the association between apoE4 and AD pathogenesis remains ambiguous. Though much of the research has focused on the ability of the apoE4 to increase the aggregation and decrease the clearance of Aβ, a lot of evidences show that apoE4 obviously impacts cholesterol transport and homeostasis in the brain [39, 89]. ApoE isoforms exert a central role in controlling the transport of brain lipid, including cholesterol, and maintaining cholesterol homeostasis in the brain [89]. Moreover, the accelerated degradation of apoE4 by astrocytes and neurons, resulting in decreased apoE4 levels in the brain [39, 89, 147]. In apoE4 mice, with the reduced secretion of apoE4 by astrocytes, Astrocytes secreted 34% less cholesterol than those from wild-type mice, the amounts of total cholesterol were significantly decreased compared with the wild-type littermates [48, 158]. ApoE4-expressing cultured astrocytes and neurons have reduced cholesterol and phospholipid secretion, decreased affinity for lipids, and increased intracellular degradation [56, 89]. In addition, in cultured neurons, cholesterol uptake is lower when the lipid is bound to apoE4 compared to apoE2 and apoE3 [56, 121]. ApoE4 is less efficient than other forms in promoting cholesterol efflux from both neurons and astrocytes [100]. Importantly, low membrane cholesterol was observed in hippocampal membranes of apoE4 AD cases [74]. To sum up, the structural differences among different apoE isoforms may account for the alterations in cholesterol trafficking. Therefore, above findings support that apoE4 markedly lead to the decrease of brain cholesterol levels in the apoE4 mice and patients, which may be involved in AD pathogenesis.

Interestingly, another direct association between cell cholesterol loss in the brain and neurodegeneration has been clearly demonstrated in Niemann–Pick C disease (NPC) [93, 150]. Niemann–Pick C disease is an autosomal recessive disorder caused by mutations in the NPC1 or NPC2 genes, which present clinical and neuropathological signs of Alzheimer's disease dementia [93, 150]. Besides, at the histological level, NPC-deficient brains present with amyloid-β deposition, neurofibrillary tangles, neuroinflammation, neuroaxonal dystrophy, and loss of neurons [93, 150]. NPC1 and NPC2 each bind to cholesterol and act in tandem in late endosomes and/or lysosomes to mediate the egress of unesterified cholesterol derived from endocytosed lipoproteins [116, 120, 150]. Thus, in NPC1- or NPC2-deficient cells, including neurons and glial cells, unesterified cholesterol and other lipids become sequestered in late endosomes and/or lysosomes [60, 93]. Accordingly, in addition to late endosomes and/or lysosomes, the amount of cholesterol in the plasma membrane, endoplasmic reticulum and axons is reduced in Npc1−/− or Npc2−/− neurons, suggesting cholesterol deficiency in the brain of Niemann–Pick C disease [60, 79, 93]. Particularly, NPC and AD share some similar molecular pathological features, including abnormal cholesterol metabolism, and involvement of amyloid-β and tau pathology [67, 93]. Obviously, further studies of similarities between AD and NPC may be useful to increase the understanding of AD pathogenesis. Taking together, the neurological deficits in NPC disease might be attributable to a deficiency, rather than an excess, of cholesterol in plasma membrane and intracellular organelles, which might be associated with AD pathogenesis [93, 150].

In addition to apoE and NPC, other genes involved in the transportation of cholesterol have been suggested as putative risk factors for AD [18, 65]. ATP-binding cassette transporters (ABC) are essential component for mediating lipid transport in brain, especially in the formation of apoE-containing lipoproteins [18, 65, 144]. Neuron and glia specific ABCA1 deficiency leads to poor lipidation of apoE, and significant decrease of cholesterol level, decrease of apoE level in brain, leading to the pathological injuries that are tightly associated with degenerative diseases neurodegenerative diseases [18, 54]. Intriguingly, many lipoprotein receptors of LDL receptor family have been identified in brain including LDLR, Low density lipoprotein receptor-related protein 1 (LRP1), very low-density lipoprotein (VLDL)-receptor, Apolipoprotein E receptor 2 (apoER2/LRP8), and the sortilin-related receptor 1 (SORL1/LP11) [18, 51]. Conditional deletion of lipoprotein receptors genes in mouse brain significantly decreases apoE and cholesterol level, resulting in related-AD neuropathological damages [51, 80, 118]. Collectively, genetic defects in the genes of ABC transporters and lipoprotein receptors of LDL receptor family are related to decrease of brain cholesterol transport and uptake, resulting in decreased brain cholesterol level. Based on the above data, we found that genetic defects in cholesterol trafficking (transport and uptake) also obviously lead to decreased brain cholesterol level which might be involved in neurodegenerative diseases, such as AD.

## Summary: brain cholesterol deficiency and AD

As stated above, a growing body of research has revealed that there is abnormal brain cholesterol metabolism in the brain in aging human and animals, SAMP8 mice, diabetic mice, FAD (5xFAD and APP/PS-1) animals, AD patients, genetic forms of AD animals and patients (ApoE4 allele, mutation of NPC1 or NPC2, polymorphism or mutations of ABC transporter and LDL receptor family). Furthermore, we found that dysregulation of cholesterol metabolism may be involved in cholesterol synthesis, trafficking and catabolism, including: 1) the decrease of de novo cholesterol synthesis; 2) and/or the decrease of cholesterol trafficking (transportation, uptake, and intracellular transportation); 3) and/or the decrease of cholesterol catabolism, suggesting the brain cholesterol loss in these different kinds of AD animals and patients (Fig. 3). Interestingly, we found that the brain cholesterol deficiency appears to be a pervasive and prominent pathological feature in these different kinds of AD models and patients (Table 1). Therefore, above data strongly suggest a new idea that there may be the brain cholesterol insufficiency or loss in the brain of AD models and patients.

**Table 1:** Defects of cholesterol metabolism in AD patients and AD models

Defects of cholesterol metabolism	Molecular changes	Evidence	References
Cholesterol synthesis	DHCR24/Seladin-1 (−)	Aβtreatment (C6, SH-SY5Y, N2A cells)	[14, 102, 135]
		chronic oxidative stress (SH-SY5Y cells)	[70]
		High glucose treatment (neuroepithelial cells, neurons)	[42, 61]
		insulin deprivation (neurons)	[61]
		diabetic rat (hippocampus, cerebral cortex)	[55, 61]
		Astrocyte-Ribotag mice and APP/PS1mice (Aged astrocytes)	[15, 106]
		AD patients (temporal lobe, hippocampus)	[45, 57, 152]
		APP/PS1 mice (cerebellum, hippocampus, cortex,)	[57, 151]
		Chronic hypoxia (hippocampus)	[88]
	HMGCR (−)	Astrocyte-Ribotag mice and APP/PS1mice (Aged astrocytes)	[15, 106]
		Diabetic rat/mouse (cerebral coretex, hypothalamus)	[124, 142]
		AD patients (hippocampus)	[152]
	SQLE (−)	Astrocyte-Ribotag mice and APP/PS1mice (Aged astrocytes)	[15, 106]
		Diabetic mice (hypothalamus)	[142]
	DHCR7 (−)	Astrocyte-Ribotag mice and APP/PS1mice (Aged astrocytes)	[15, 106]
		diabetic mice (hypothalamus)	[142]
	SREBF2 (−)	Aged cortical astrocytes	[107]
		Diabetic rat/mouse (cerebral cortex, hypothalamus)	[124, 142]
Cholesterol trafficking	APOE (+)	Astrocyte-Ribotag mice (Aged astrocytes)	[15]
		APOE4 knock-in mice	[48]
		5xFAD mice	[110]
		Diabetic rat (cerebral cortex)	[124]
		APP/PS1 mice (hippocampus)	[151]
	ABC transporters (ABCA1/G1/C1)(+)	Diabetic rat (cerebral cortex)	[124]
		5xFAD mice	[110, 156]
		APP/PS1 mice	[151]
	NPC (−)	NPC-deficient cells	[60, 79]
	LDLR (−)	LRP1-deficient neurons/LRP1 knock-out mice	[80]
		Diabetic rat (cerebral cortex)	[124]
Cholesterol catabolism	CYP46A1 (−)	AD patients (entorhinal cortex)	[152]
Cholesterol deficiency (uncategorized)		AD patients (lipid rafts from frontal cortices and entorhinal cortex, temporal cortex)	[34, 101]
		Aged mice (lipid rafts from neocortex)	[35]
		APP/PS1 mice (lipid rafts from neocortex)	[35]
		AD patients (CSF, temporal gyrus, white matter)	[68, 96, 123]
		Aged human (frontal and temporal cortices)	[137, 143]
		APOE mice (primary astrocyte)	[158]

− Expression of specific gene is downregulated, + expression of specific gene is upregulated

## Widen the view on AD: brain cholesterol level and AD

Over the last decades, increasing biochemical and molecular biological evidences reveal that altered cholesterol metabolism appears to play fundamental roles in amyloid plaque formation, tau hyperphosphorylation, synaptic loss, and apoptosis, suggesting a key role of cholesterol in the initiation and progression of AD [1, 5, 21, 74, 75, 91, 93, 115]. However, the role of cholesterol for neurodegeneration such as AD, remains still controversial [1, 5, 81, 93, 115, 154].

### High plasma cholesterol level and AD

Early epidemiological studies suggest that increased level of plasma cholesterol is a risk factor for the development of AD [77, 140, 149]. Several studies show that lipophilic statins (brain-permeant statin) which can cross the blood–brain barrier, present a reduced incidence of AD [7, 37, 38, 59]. However, randomized double-blind placebo-controlled studies have shown no beneficial effect of statins on the progression of symptoms in subjects with AD [126, 134]. On the contrary, the lipophilic statins induce high amyloid production and senile plaque deposition in mice brain [74, 109]. And recent studies show that lipophilic statins could increase the risk of developing dementia, coupled with the decrease of brain cholesterol level, suggesting brain cholesterol loss have the increasing risk of dementia [47, 108]. Because the blood–brain barrier prevents entry of cholesterol-rich lipoproteins, all cholesterol in the brain is made locally. Thus, causal correlations between high blood cholesterol and AD are controversial.

### High brain cholesterol level and AD

Early-study shows that brain cholesterol is high in the brains of patients with AD [25]. However, many studies suggest that cholesterol levels don’t differ in hippocampal region and the cerebral cortex tissue of AD patients compared with control subjects [32, 52]. Further, there are inconsistent outcomes in brain cholesterol levels of AD patients, but the variability of cholesterol level amongst the studies might obviously pertain to brain tissue sample selection, tissue preparation and assay methods [153]. Consequently, there is no enough evidence to prove that high brain cholesterol level contributes to AD.

### Low brain cholesterol level and AD

Based on the data in the part 5, increasing evidences support that there may be obvious cholesterol loss in the brain of different kinds of AD animals and patients. Intriguingly, we found that the different kinds of AD models and patients included the major risk factors for AD, such as Aβ, aging, diabetes and diabetes-related factors, oxidative stress, chronic inflammation, and genetic risk factors, metabolic syndrome, etc. (Fig. 3). Furthermore, the brain cholesterol loss seems to be tightly associated with major risk factors from the different kinds of AD model animals and patients. Thus, we suppose that the brain cholesterol loss seems to be induced by the major risk factors for AD in the different kinds of AD models and patients. Thus, accumulating evidences suggest that there may be a direct link between brain cholesterol loss and major risk factors for AD.

Although there are conflicting reports on the role of cholesterol in AD, it is not difficult to envision how reduced neuronal cholesterol levels can lead to the neuropathological impairments which are associated with AD, resulting in the brain dysfunction [16, 27, 33, 40, 49, 53]. Very importantly, increasing evidences reveal that the neuronal cholesterol deficit/loss could induce the disruption of membrane lipid rafts and/or intracellular organelles, and eventually leads to the formation of pathological impairments, which are obviously linked to the pathological changes which are associated with the pathogenesis of AD and other neurodegenerative diseases [1, 5, 9, 16, 21, 24, 27, 33, 36, 40, 44, 74, 75, 91, 93, 99, 115, 119]. Taking together, compelling evidences suggest that the brain cholesterol deficiency could contribute to AD pathogenesis.

## Conclusion and future perspective

In this paper, we try to provide a current state of research on the role of DHCR24 in the pathogenesis of Alzheimer's disease. Importantly, based on previous studies and our research on DHCR24, the decreased cholesterol level by DHCR24 knockdown could contribute to neurodegenerative diseases such as AD, thus, these findings suggest that augmentation of DHCR24 in the affected brain areas might provide a potential therapeutic approach to intervene in AD and other neurodegenerative diseases. As a key node in the control of cholesterol synthesis and homeostasis, targeting DHCR24, careful modulation of brain cholesterol metabolism may provide an alternative or complementary interventional approach in order to test whether modulating brain neuronal cholesterol metabolism is a viable strategy for preventing AD.

With the continuously growing body of knowledge in this field, a body of studies has pinpointed that brain cholesterol deficiency is very likely to be an early and common driving factor in the onset and development of AD, and seems to be intimately linked with the generation of amyloid plaques, tauopathy, synaptic injuries, neuronal loss, which are central to the pathogenesis of AD. To sum up, based on previous data and research on DHCR24, we suppose that the brain cholesterol deficiency/loss could trigger the onset and progression of AD (Fig. 3). In addition, although there are many acceptable hypotheses, such as amyloid-β, tau, and inflammatory hypotheses, the pathogenetic mechanism of Alzheimer's disease is still elusive. Furthermore, uncovering the key causative alterations of AD can be valuable in developing models for AD treatment. In order to gain a better understanding of cholesterol’s role in AD pathogenesis, we hope that this new proposal will stimulate further experimental research in this direction that allows the testing of our hypothesis. In the review, we only chose some topics for in depth discussions. Unfortunately, a lot of important research topics were left with little or with no discussion. Certainly, in order to gain a more comprehensive recognition of cholesterol’s role in AD pathogenesis, we still need to investigate more the role of cholesterol metabolism in AD patient and animal brains, including the brain cholesterol amount, specific regional changes, as well as the distribution of cholesterol within neurons such as lipid rafts or intracellular organelles.

## Data Availability

Not applicable.

## Abbreviations

5xFAD: Five familial AD
7-DHC: 7-Dehydrocholesterol
AD: Alzheimer’s disease
Akt: Protein kinase
APP/PS1: APPswe/PS1deltaE9
APP: Aamyloid-β precursor protein
Aβ: Amyloid-β protein
BACE1: Beta-site amyloid precursor protein cleaving enzyme 1 or β-secretase
DHCR24: 3β-Hydroxysterol-Δ24 reductase or 24-dehydrocholesterol reductase
DHCR7: 7-Dehydrocholesterol reductase
DM: Diabetes mellitus
DRM: Detergent-resistant membrane domain
FAD: Familial Alzheimer's disease
GGA3: Golgi-localized gamma-ear-containing ARF binding protein 3
GSK3β: Glycogen synthase kinases-3beta
HMGCR: Hydroxy-3-methylglutaryl-CoA reductase
IGFs: Insulin-like growth factors
KO: Knockout
K–R pathway: Kandutsch–Russell pathway
LDM: CYP51A1 or Lanosterol 14-a-demethylase
LOAD: Late-Onset Alzheimer's Disease
mTOR: Mammalian target of rapamycin
NGF: Nerve growth factor
PI3-K: Phosphoinositide 3-kinase
PP2A: Protein phosphatase 2A
SAD: Sporadic AD
Seladin-1: Selective Alzheimer’s disease indicator 1
shRNA: Short hairpin RNA
SQLE: Squalene epoxidase
STZ: Streptozotocine
Tau: Microtubule-associated protein tau
TH: Thyroid hormones
